# One Species, Three Pleistocene Evolutionary Histories: Phylogeography of the Italian Crested Newt, *Triturus carnifex*

**DOI:** 10.1371/journal.pone.0041754

**Published:** 2012-07-26

**Authors:** Daniele Canestrelli, Daniele Salvi, Michela Maura, Marco A. Bologna, Giuseppe Nascetti

**Affiliations:** 1 Dipartimento di Scienze Ecologiche e Biologiche, Università della Tuscia, Viterbo, Italy; 2 Centro de Investigação em Biodiversidade e Recursos Genéticos, Vairão, Portugal; 3 Dipartimento di Biologia Ambientale, Università Roma Tre, Rome, Italy; Ecole Normale Supérieure de Lyon, France

## Abstract

Phylogeographic patterns of temperate species from the Mediterranean peninsulas have been investigated intensively. Nevertheless, as more phylogeographies become available, either unique patterns or new lines of concordance continue to emerge, providing new insights on the evolution of regional biotas. Here, we investigated the phylogeography and evolutionary history of the Italian crested newt, *Triturus carnifex*, through phylogenetic, molecular dating and population structure analyses of two mitochondrial gene fragments (ND2 and ND4; overall 1273 bp). We found three main mtDNA lineages having parapatric distribution and estimated divergence times between Late Pliocene and Early Pleistocene. One lineage (S) was widespread south of the northern Apennine chain and was further geographically structured into five sublineages, likely of Middle Pleistocene origin. The second lineage (C) was widespread throughout the Padano–Venetian plain and did not show a clear phylogeographic structure. The third lineage (N) was observed in only two populations located on western Croatia/Slovenia. Results of analysis of molecular variance suggested that partitioning populations according to the geographic distribution of these lineages and sublineages explains 76% of the observed genetic variation. The phylogeographic structure observed within *T. carnifex* and divergence time estimates among its lineages, suggest that responses to Pleistocene environmental changes in this single species have been as diverse as those found previously among several codistributed temperate species combined. Consistent with the landscape heterogeneity, physiographic features, and palaeogeographical evolution of its distribution range, these responses encompass multiple refugia along the Apennine chain, lowland refugia in large peri-coastal plains, and a ‘cryptic’ northern refugium.

## Introduction

Identifying Pleistocene refugia is a central task of phylogeographical research ([1]–[3] and references therein), and ongoing climate change has led to increased interest in the identification and characterization of these areas [4]. Glacial refugia allowed species to survive during unfavourable climatic phases of the Pleistocene and are often hotspots of current intraspecific diversity. Recent studies have shown that a plethora of microevolutionary processes encompassing demographic size variations, population fragmentations, secondary contacts, and population admixture acted in glacial refugia and contributed to shape the high refugial genetic diversity [3], [5]–[12]. Therefore, glacial refugia are important both for long-term preservation of species and for their evolutionary potential. Moreover, evidence supporting the importance of genetic diversity with respect to community structure and ecosystem resilience is growing [13], [14]. Thus, assessing the geographic location of glacial refugia and understanding what microevolutionary processes were involved in the formation and long-term maintenance of genetic diversity hotspots in these areas have crucial relevance with respect to conservation at multiple levels of biodiversity [4], [15], [16]. Interestingly, in many regions worldwide, glacial refugia for temperate species have been identified in coastal lowlands (e.g. [17]–[20]) and/or at the so-called ‘rear edges’, the current low-latitude margins of species' ranges (e.g. [21]–[24]). Populations in these areas are expected to be especially threatened by climate change, rising special concerns for their long-term conservation (see [4], [15], [25], [26] for a thorough discussion of the genetic consequences of recent climate change).

The western Mediterranean region has been the subject of intensive phylogeographical efforts [5], [6], [27], [28]. The Balkan, Iberian, and Italian peninsulas have long been identified as the most important refugial areas in the region, but as more studies were performed, increasingly complicated patterns appeared. For example, within these three peninsulas, a scenario of multiple refugia is emerging for a growing number of species (see [6], and references therein). While in many cases long-term demographic stability can explain the occurrence of intraspecific diversity hotspots in these areas, in many others repeated cycles of population fragmentation and secondary admixture were involved (e.g. [10], [11], [29], [30]). Moreover, peri-glacial areas and coastal lowlands exposed by glaciation-induced sea-level lowstands are emerging as important, previously underrated refugia, which allowed some species to overcome the negative demographic effects of glacial climate [19]. On the whole, these studies reveal that the species' responses to Pleistocene climatic oscillations were even more diverse than previously thought [5], [7], [19], [31]–[35].

**Figure 1:**
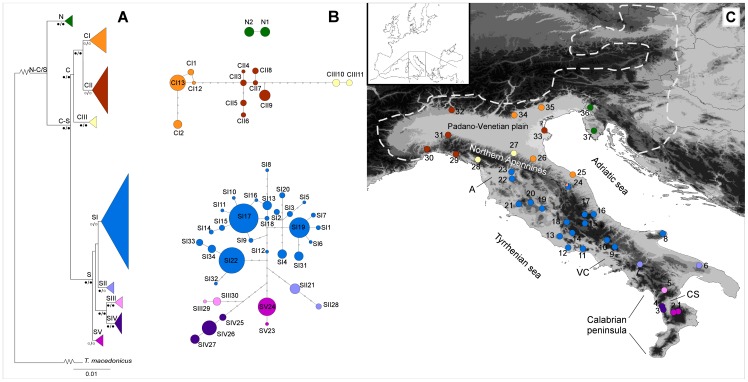
Geographic distribution of the 37 sampling sites and phylogenetic relationships of the 49 haplotypes found in *Triturus carnifex*. A, Maximum likelihood (ML) tree showing the phylogenetic relationships among the 49 haplotypes found in *Triturus carnifex*. Terminal haplogroups were collapsed. Clade names and bootstrap (bs) values of ML and Maximum parsimony (MP) trees (ML/MP), respectively, are shown above and below each node (grey circles: bs >70%; black circles: bs >85%). B, Statistical parsimony networks, with haplotypes numbered as in Table 1. Circles size is proportional to haplotype frequency; open dots represent missing intermediate haplotypes. C, Geographical distribution of the 37 populations sampled. Populations (shown as pie diagrams) are coloured according to the main haplogroups in panels A and B. White dotted line shows the northern edge of the species' distribution; A, Arno river basin, CS, Crati–Sibari plain, VC, Volturno–Calore river drainage basin. Inset: Geographical location of the study area within the western Palearctic region.

In this study, we examined the phylogeography of the Italian crested newt, *Triturus carnifex*. This amphibian belongs to the *T. cristatus* superspecies, a species group distributed in Europe and western Asia (reviewed in [36]). *T. carnifex* is common and widespread from sea level to about 1000 m a.s.l. ([37], but see also [38]) where it breeds in a wide range of freshwater habitats [38]–[40]. *T. carnifex* is generally considered a poor disperser with short migration distances, generally less than 1 Km/year ([36], [41]; but see also [42]). It is mainly distributed in the Italian peninsula south of the Alpine arc, reaching East Slovenia, northern Croatia and Austria ([36]; Figure 1C). Interestingly, it is absent in most of the central and southern Calabria, an area that has been repeatedly indicated as the most important glacial refugium for temperate species in the Italian peninsula (see [10], [11], [43]; and references therein).

Previous studies based on allozyme markers [44], [45] suggested that the genetic diversity of *T. carnifex* populations is geographically structured into two population groups distributed north and south of the north-central Apennine chain. Thus, both the geographic pattern of distribution and early evidence of population structure suggest that the Plio-Pleistocene evolutionary history of *T. carnifex* could be unique, not conforming to the scenario inferred for most temperate species along the Italian peninsula, i.e. a (more or less) fragmented glacial range encompassing the Calabrian region (the main refugium in southern Italy) and a postglacial northward expansion along the peninsula (e.g. [21], [46]). Therefore, in this study we carry out phylogenetic, molecular dating and population structure analyses in order to investigate the evolutionary history of this newt. Finally, through a comparison with previous studies of co-distributed species, we put in evidence novel patterns of phylogeographic concordance and their significance for the evolution of regional biotas.

## Materials and Methods

### Ethics statement

The Italian Ministry of Environment approved all animal procedures in this study, including capture, handling, and tissue sampling (DPN/2D/2003/2267). Since the study did not involve laboratory work on living animals, authorization from the Ministry of Health was not required. Newts were captured with nets at breeding ponds. We collected tissue samples from tail tips, anaesthetizing newts by submerging them in a 0.1% solution of MS222 (3-aminobenzoic acid ethyl ester). Immediately after the completion of the procedure, tissue samples were stored in 96% ethanol and all newts were released at the collection site. No newts were brought to the laboratory and no newts were sacrificed. All sampling took place in public areas and no additional permits or approvals were required for these sites.

### Sampling and laboratory procedures

A total of 231 *Triturus carnifex* individuals were sampled from 37 localities spanning the species' range. Detailed information about sampling localities and number of individuals sampled in each locality are shown in Table 1 and Figure 1C. DNA extraction was performed by following the standard cetyltrimethyl ammonium bromide (CTAB) protocol [47]. Two mitochondrial fragments were amplified and sequenced for all individuals. One fragment comprised part of the NADH dehydrogenase subunit 4 gene and the tRNA^His^ gene (hereafter referred to as ND4), and the other fragment comprised the NADH dehydrogenase subunit 2 gene (hereafter referred to as ND2). Preliminary amplifications and sequencing of the ND4 fragment were performed using primers ND4 and LEU [48], and then the internal primers ND4carnF1 (ACCCCATTAACAAAAGAAATAGCA) and ND4carnR2 (GTGTTTCATAACTCTTCTTGGTGTG) were designed and used to screen all individuals. Preliminary amplifications and sequencing of the ND2 fragment were performed using primers H5018 and L3780 [49], and then the internal primer TCND2F2 (TCCTTGCTTGAATAGGACTAGAAAT) was designed and used in conjunction with H5018 to screen all individuals.

**Table 1:** Geographic location, number of individuals (n) and haplotype composition of the 37 populations sampled of *Triturus carnifex*.

Population	Country	Locality	Latitude (N)	Longitude (E)	n	Haplotypes (n)
1	Italy	Cecita	39°22′	16°30′	**6**	**SV23 (1), SV24 (5)**
2		San Pietro in Guarano	39°20′	16°21′	**9**	**SV24 (9)**
3		Laghicello	39°25′	16°5′	**6**	**SIV26 (6)**
4		Due Uomini	39°33′	16°1′	**15**	**SIV25 (4), SIV26 (5), SIV27 (6)**
5		Taverna Magnano	40°03′	16°07′	**6**	**SIII29 (1), SIII30 (5)**
6		Alberobello	40°49′	17°14′	**3**	**SII28 (3)**
7		Conza	40°52′	15°18′	**7**	**SII21 (7)**
8		Torre Palermo	41°50′	16°2′	**15**	**SI22 (15)**
9		Sepino	41°23′	14°34′	**1**	**SI32 (1)**
10		Sessano	41°37′	14°20′	**9**	**SI33 (4), SI34 (5)**
11		Campo di Mele	41°23′	13°31′	**7**	**SI12 (1), SI13 (6)**
12		Circeo	41°20′	13°2′	**5**	**SI14 (2), SI15 (3)**
13		Doganella	41°45′	12°47′	**7**	**SI17 (1), SI19 (6)**
14		Jenne	41°52′	13°11′	**7**	**SI16 (1), SI17 (4), SI18 (2)**
15		Rocca di mezzo	42°9′	13°38′	**5**	**SI9 (2), SI10 (1), SI17 (2)**
16		San Quirico	42°26′	13°50′	**7**	**SI8 (1), SI22 (6)**
17		Campo Imperatore	42°25′	13°37′	**7**	**SI17 (7)**
18		Navegna	42°9′	13°2′	**11**	**SI11 (1), SI17 (10)**
19		Alviano	42°37′	12°14′	**7**	**SI6 (1), SI7 (2), SI19 (3), SI20 (1)**
20		Rufeno	42°46′	11°53′	**2**	**SI19 (1), SI20 (1)**
21		Roccalbegna	42°44′	11°30′	**8**	**SI1 (2), SI19 (5), SI20 (1)**
22		Greve in Chianti	43°35′	11°18′	**8**	**SI3 (1), SI4 (6), SI5 (1)**
23		Firenze	43°45′	11°15′	**6**	**SI2 (3), SI3 (2), SI19 (1)**
24		San Severino Marche	43°18′	13°4′	**8**	**CI13 (2), SI31 (6)**
25		Senigallia	43°41′	13°12′	**5**	**CI12 (1), CI13 (4)**
26		Terra del sole	44°11′	11°57′	**5**	**CI13 (5)**
27		Mulino di Pianoro	44°21′	11°19′	**5**	**CIII10 (2), CIII11 (3)**
28		Minucciano	44°10′	10°12′	**2**	**CIII10 (2)**
29		Stagno Bargone	44°19′	9°29′	**5**	**CII5 (3), CII6 (2)**
30		Piampaludo	44°27′	8°34′	**2**	**CII9 (2)**
31		Donega	44°56′	9°14′	**5**	**CII7 (2), CII8 (3)**
32		Montevecchia	45°43′	9°23′	**5**	**CII9 (5)**
33		Porto Caleri	45°5′	12°19′	**4**	**CII3 (3), CII4 (1)**
34		Le Poscole	45°36′	11°23′	**5**	**CI2 (5)**
35		Busa de San Piero	45°48′	12°08′	**3**	**CI1 (3)**
36	Slovenia	Komen	45°49′	13°45′	**5**	**N1 (5)**
37	Croatia	Svetvinčenat	45°4′	13°52′	**5**	**N2 (5)**

Amplifications were performed in a 25 µl volume containing MgCl2 (2.5 mM), reaction buffer (5×, Promega), the four dNTPs (0.2 mM each), the two primers (0.2 µM each), the enzyme Taq polymerase (1 U, Promega) and 2 µl of DNA template. Polymerase chain reaction (PCR) was performed with a step at 95°C for 5 min followed by 30 (ND4) or 35 (ND2) cycles of: 94°C for 1 min, 56°C for 1 min, 72°C for 1 min, and a single final step at 72°C for 10 min. Purification and sequencing of the PCR products were carried out by Macrogen Inc. (www.macrogen.com) by using an ABI PRISM 3700 sequencing system. All sequences were deposited in GenBank (accession numbers: JQ598071–JQ598166).

### Data analysis

Electropherograms were visually checked using FinchTv 1.4.0 [50] and aligned using Clustal ×2.0 [51]. MEGA5 [52] was used to analyse sequence variation.

Phylogenetic relationships among *T. carnifex* haplotypes were inferred using Maximum Likelihood (ML) and Maximum Parsimony (MP) methods. For these analyses, the closely related species *T. macedonicus* was used as outgroup (GenBank accession number NC015794).

The best-fit model of nucleotide substitution for our dataset was selected among 88 alternative models using the Akaike Information Criterion (AIC, [53]) implemented in jModelTest 0.1.1 [54]. We first analysed the ND4 and ND2 fragments separately, and then combined. TIM1+Γ [55] was the best fit model in all cases. Consequently, the combined dataset (with the gamma distribution shape parameter = 0.10) was used in all subsequent analyses.

ML analyses were performed with PhyML 3.0 [56]. Tree topologies were estimated using the SPR&NNI option, which performs both the available methods (i.e the Nearest Neighbor Interchanges (NNI), and the Subtree Pruning and Regrafting (SPR)) and returns the best solution among the two. MP analysis was computed using PAUP [57], with all characters equally weighted and unordered. A heuristic search was carried out, with tree bisection and reconnection (TBR) branch swapping and 10 rounds of random sequence addition. The robustness of the inferred ML and MP tree topologies was assessed by the non-parametric bootstrap method with 1000 replicates.

Phylogenetic relationships among haplotypes were also inferred by the statistical parsimony procedure for phylogenetic network estimations [58] by using the software TCS 1.2.1 [59].

Time to the most recent common ancestor (TMRCA) of the main mtDNA lineages was estimated by using the distance-based least squares (LS) methods recently described by Xia & Yang [60] and implemented in the software DAMBE [61]. The hypothesis of clock-like evolution of our sequences was assessed by performing a likelihood ratio test in DAMBE. This test did not reject the molecular clock hypothesis for our dataset. To specify a tree topology we used the ML tree previously estimated by PhyML. The divergence between *T. carnifex* and *T. macedonicus* was used to set a calibration point. According to Arntzen et al. [62], this divergence was estimated to date back to the end of the Messinian salinity crisis and the consequent reflooding of the Adriatic Sea (5.337 million years ago (Ma)). Finally, to perform the LS analysis in DAMBE we set the ‘softbound’ option and ‘MLCompositeTN93’ genetic distance, as suggested by Xia & Yang [60], along with 1000 bootstrap re-samplings to obtain standard deviations of the time estimates.

To understand how genetic variance was hierarchically distributed among groups, among populations within groups, and within populations, we performed the analysis of molecular variance (AMOVA) by using Arlequin 3.5.1.2 [63]. Groups were defined a priori, according to the main geographic discontinuities in the distribution of genetic variation, as defined by previous phylogenetic analyses. The analysis was run using the Tamura & Nei model (TrN+Γ [64]), which is the best approximation of the TIM1+Γ model available in ARLEQUIN. The significance of the variance components and fixation indices was tested using 10100 permutations.

To assess the occurrence of a significant pattern of isolation-by-distance, the correlation between geographic and genetic distances separating populations was evaluated using Mantel tests with the software ZT [65]. Following suggestions by Rousset [66], geographic distances were log-transformed, and genetic distances were estimated as the mean distances among populations calculated with MEGA by using the TrN+Γ model. Mantel tests were performed for the entire data set and for each main clade defined by previous phylogenetic analyses, along 1000 bootstrap replicates.

## Results

For all individuals analysed the ND4 fragment was 638 bp in length, comprising 563 bp of the (3′) NADH dehydrogenase subunit 4 gene and 75 bp of the tRNA^His^ gene, and the ND2 fragment was 635 bp. The combined dataset (overall 1273 bp) included 128 variable positions, of which 70 were parsimony informative. We did not find indels or stop codons within the coding region of either the ND2 or the ND4 fragments. A total of 49 haplotypes were found in the combined fragment, and their geographic distribution is presented in Table 1.

The tree obtained by the ML method is shown in Figure 1A. The log-likelihood score for the ML tree was −3853.93018. MP analysis yielded 1324 most parsimonious trees of 186 steps in length (consistency index = 0.715; retention index = 0.869). Tree topologies were identical between MP and ML trees at main nodes, with minor differences at some terminal nodes. Three main clades were found, and their geographic distribution among populations is shown in Figure 1C. One clade (referred to as clade N) included only two haplotypes and was geographically restricted to north-eastern samples 36 and 37. The second clade (referred to as clade C) was found among samples from the Padano–Venetian plain and northwestern Apennines (samples 24–35), and the third clade (referred to as clade S) was widespread throughout the reminder of the species' range along the Italian peninsula (samples 1–24). Average Tamura–Nei sequence divergence among the three clades was 0.029 (standard error (SE) 0.006) for the clade pairs N–C and N–S, and 0.021 (0.004 SE) between clades C and S. Co-occurrence among these main clades was observed only in sample 24 (clades C and S). Three main subclades (referred to as CI, CII, and CIII) were observed within clade C, but they showed no clear geographic pattern of distribution (Figure 1 and Table 1). Instead, five subclades within clade S showed a clear geographic association. Subclades SIII, SIV, and SV were restricted in the Calabrian peninsula (samples 5, 3–4, and 1–2, respectively). Subclade SII (samples 6–7) was distributed north of this area to the Volturno–Calore basin. Finally, subclade SI (the most frequent in the dataset) was widespread throughout the remainder of the Italian peninsula. All of the above clades and subclades were supported by high bootstrap values (>70%).

Phylogenetic networks among the haplotypes found are shown in Figure 1B. Under the 95% criterion for a parsimonious connection, three distinct networks were generated (N, C, and S), and they corresponded to the three main clades of the phylogenetic trees. The haplotypes of networks C and S formed three and five subgroups, respectively, clearly corresponding to the subclades yielded by the tree-building methods.

**Figure 2:**
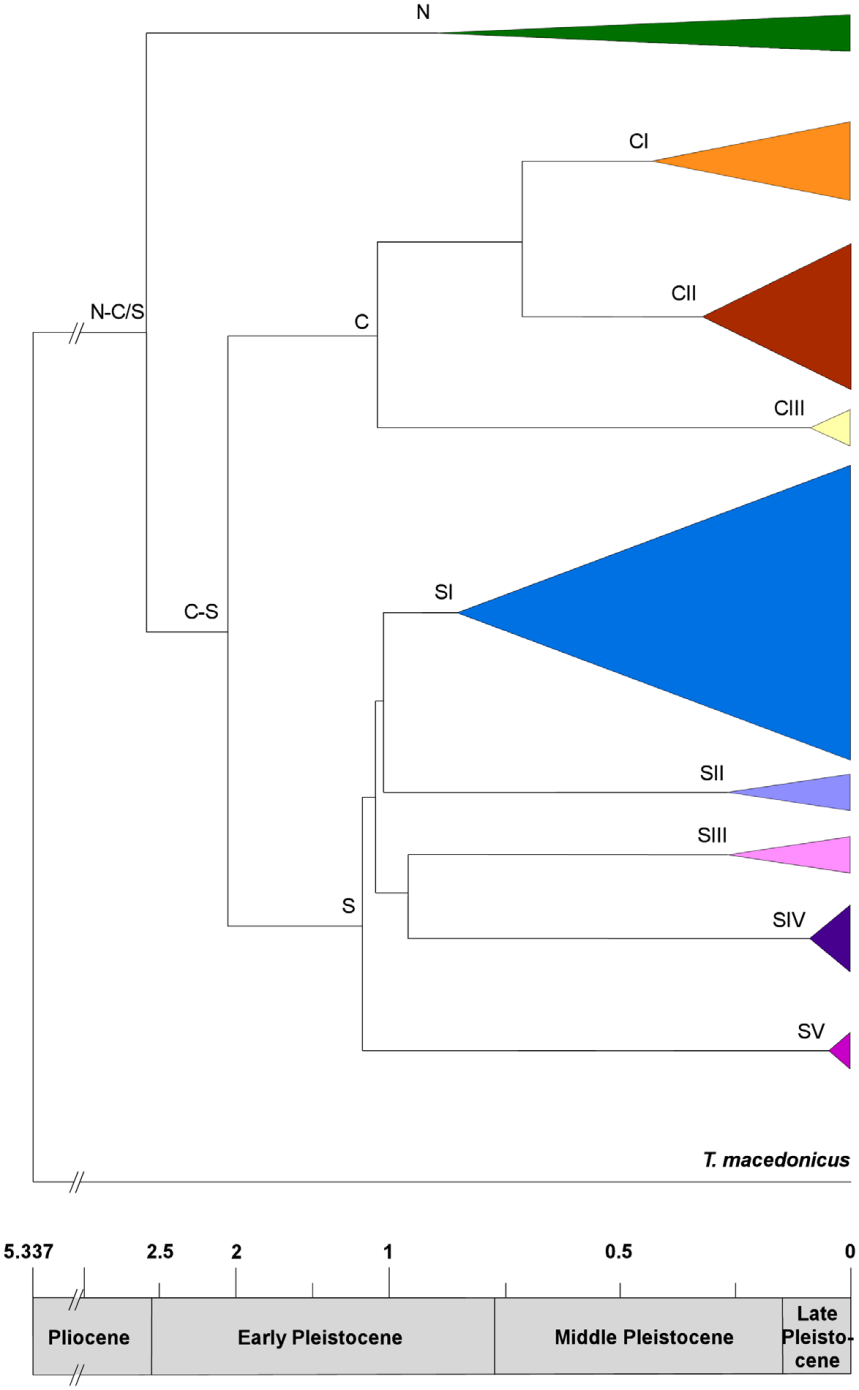
Chronogram of the main mtDNA lineeages found in *Triturus carnifex.* Chronogram showing the estimated times to the most recent common ancestor (TMRCA) for the main mtDNA lineages of *Triturus carnifex*. The calibration point (5.337) and the ranges of the main historical epochs on the scale bar are reported in million years. Clades were named as in Figure 1A.

TMRCA estimates for the main mtDNA lineages are shown in the chronogram of Figure 2. The TMRCA for the entire ingroup was estimated to have occurred between the Late Pliocene and Early Pleistocene (2.619±0.426 Ma), and the divergence between clades C and S fell well within the Early Pleistocene (2.049±0.364 Ma). Finally, most of the splits within these clades likely occurred late in the Early Pleistocene.

For the AMOVA analysis the following seven groups were defined (see Figure 1): (1–2), (3–4), (5), (6–7), (8–24), (25–35), (36–37). Since the three subclades found within clade C did not show a clear geographic structure (see above), all individuals carrying haplotypes from clade C were assigned to a single group (25–35). This analysis showed that 70.59% of the overall genetic variance can be attributed to differences between groups, 24.40% to differences among populations within groups, and 5.01% to differences within populations. All the covariance components were highly significant (Table 2).

**Table 2:** Summary of the molecular variance analyses, with populations grouped according to the phylogenetic results.

Source of variation	Degree of freedom	Percentage of variation*	Fixation Indices*
Among groups	6	70.59*	*F*_ct_ = 0.70*
Among populations within groups	30	24.40*	*F*_sc_ = 0.83*
Within populations	191	5.01*	*F*_st_ = 0.95*

* P<0.001.

The Mantel tests performed between genetic distances and log-geographic distances suggested the occurrence of a statistically significant (*P*<0.01) but weak pattern of isolation by distance, both within the entire dataset (*R* ^2^ = 0.29) and within the range of clade S (*R* ^2^ = 0.31). No significant pattern of isolation by distance was detected among the populations of clade C.

## Discussion

Our results revealed an unexpected phylogeographic pattern compared with previous studies of the genetic structure among *T. carnifex* populations. Indeed, analyses of allozyme genetic variation carried out by Scillitani & Picariello [44] and Arntzen [45], consistently identified two main lineages, roughly distributed in northern and peninsular Italy respectively. In addition, the study of the mtDNA variation within the *T. cristatus* species group by Arntzen et al. [62] identified two main lineages within the range of *T. carnifex*, one found among samples from peninsular Italy and the other among samples located east and north of the Alpine arc. In contrast, our results revealed the occurrence of three main mtDNA lineages (clades N, C and S; Figure 1), which apparently contrasts with the results of those previous studies. Nevertheless, when the respective sampling schemes are compared, each of those previous studies may have overlooked samples within the range of one of the three lineages. Both allozyme studies were based on samples collected south of the Alpine arc, and thus, lacked samples from the putative range of our clade N, while the study by Arntzen et al. [62] lacked samples from the Padano–Venetian plain, i.e. the putative range of our clade C.

### Divergence and secondary contact among main lineages

The divergence among the three lineages was roughly estimated to have occurred from the Plio-Pleistocene boundary to early in the Lower Pleistocene (Figure 2). Palaeoenvironmental changes were profound during this epoch and have been studied intensively in the Mediterranean region [67]–[69]. Climate became cooler and dryer, and the prevailing features of today's climate, including marked seasonality, became established at this time [70]–[75]. Accordingly, pollen spectra and fossil data, indicate substantial changes in the distributions and assemblages of temperate species [69], [70], [76]–[78]. It is likely that *T. carnifex* experienced major distributional changes during this period, including major fragmentations into three lineages, as suggested by its current genetic structure (our data; [44], [45], [62]), at least under the assumption of niche conservatism.

The phylogeographic breaks found by us among the main lineages of *T. carnifex* closely match prominent biogeographic discontinuities along the Italian peninsula (see [6]).

The geographic area where lineages C and N have been found in close contiguity falls on the north-eastern side of the Padano–Venetian plain. Although outside this area we did not detect any admixed populations where the two lineages co-occur, the continuous distribution of *T. carnifex* across this phylogeographic discontinuity strongly suggests the existence of a secondary contact zone between lineages C and N. Interestingly, this geographic area not only marks the range boundaries of many species, but has also been indicated as an effective biogeographical crossroad between the Dinaric and Italian districts for many taxa and as a site of clustering of contact zones and hybrid zones between both intraspecific lineages and closely related species (this study; [3], [49], [79]–[86]).

The southern edge of the lineage C range is delimited by a well-known biogeographic barrier, the northern Apennines. Lineage C extends narrowly to the south at both the eastern and western sides of this mountain area, and at least on the eastern side, it establishes a secondary contact zone with lineage S, as revealed by the co-occurrence of haplotypes from both lineages in our single sample 24. Clusters of species' range edges, phylogeographic breaks, and contact zones along the northern Apennines have similarly been found in several temperate species, including amphibians [87]–[90].

The clustering of phylogeographic breaks, secondary contact zones, and species' range edges identifies both the north-eastern side of the Padano–Venetian plain and the northern Apennines as suture zones (under the extended definition by [91]). This has several significant implications. First, the phylogeographic concordance between the pattern observed in *T. carnifex* and those previously found in several other species is strong evidence in favour of the historical rather than stochastic origin of the observed discontinuities [92]–[94]. Second, suture zones offer unique opportunities to compare levels and patterns of gene exchange in relation to divergence history and phenotypic evolution of different taxa [3], [95]. In the western Palearctic region, such opportunities have been especially exploited for the northern and central portion of the region (e.g. [3], [81], [96]), whereas limited research has been devoted to cases in the Mediterranean region (but see [7], [10], and references therein). The two zones underscored here are located in key areas of the western Palearctic region and are transitional between peninsular Italy and the continent (northern Apennines), and between the Italian and the Balkan regions (north-eastern side of the Padano–Venetian plain). Thus, future studies in these areas could help to gain deeper insights on the evolutionary history of the Mediterranean hotspot of biodiversity. Finally, because these zones are hotspots of divergence and evolutionary potential, they also merit special consideration with respect to biodiversity conservation (see [16]).

### One species, three Pleistocene evolutionary histories I: Northern ‘cryptic’ refugia

The geographic distribution of the three lineages (N, C, and S), their estimated divergence times, and their intra-lineage phylogeographic structures suggest that they have had independent and substantially different evolutionary histories throughout most of the Pleistocene.

Lineage N was observed only at the easternmost portion of the species range (sites 36–37). This observation is also confirmed by a comparison of previously published sequence data [97], [98] with our data. In fact, when our haplotypes N1 and N2 are compared with the more eastern and southern samples from these previous studies (one sequence from Sinac, Croatia and one sequence from Kramplje, Slovenia; Genbank accessions: GQ258936, GQ258952; GU982385, GU982459), average sequence divergence never exceeded 0.009, that is well below the value we found among haplotypes belonging to clades C and N (0.021, 0.004 SE; data available upon request). Although more samples east of the Alpine chain clearly will be needed before inferring the Pleistocene evolutionary history of lineage N, data currently available suggest long-term isolation of this lineage in western Croatia/Slovenia. The occurrence of lineage C in the Padano–Venetian plain, *T. macedonicus* in the Balkans, *T. cristatus* in central Europe, and *T. dobrogicus* in eastern Europe (see [36]), make neighbouring areas, including the Italian and the Balkan peninsulas, less plausible as long-term refugia for this lineage. Multiple lines of evidence suggest the existence of northern (cryptic) refugia for temperate species [2], [31], areas of sheltered topography that provided suitable microclimates for the survival of thermophilous species outside the traditional southern peninsular refugia. This hypothesis has recently received support from several phylogeographic studies of various temperate organisms, including plants and animals (see [2] for a review). In the western Croatia/Slovenia area, the occurrence of ancient and divergent lineages of *T. carnifex* echoes previous findings from several phylogeographic, palaeobotanical and palaeoclimatic studies [84], [99]–[101], thus indicating the prominent contribution of this northern refugium to the present-day genetic pools of many temperate species.

### One species, three Pleistocene evolutionary histories II: ‘Refugia-between-refugia’

The occurrence of clade C in the Padano–Venetian plain and its deep divergence with clades N and S provide strong evidence in support of a long-term persistence of this lineage in this area and suggest that the Padano–Venetian plain could have acted as a long-term refugium for *T. carnifex* over multiple Pleistocene glaciations. The occurrence of closely related lineages around the range of the Padano–Venetian lineage would disprove the alternative hypothesis of a recent (re)colonization of this region from neighbouring areas, including north and west of the Alpine arc and the eastern coast of the Adriatic Sea, i.e. the distribution areas of the closely related species *T. cristatus* and *T. macedonicus*. Palaeogeographic, sedimentological, and palaeontological (fossil and pollen) data have shown that following the south-eastern widening of the Padano–Venetian plain due to glaciation-induced marine regressions [102]–[104], a vast alluvial plain environment was established in this area [105]. This provided a paleoenvironmental scenario suitable for the survival of temperate species, particularly amphibians, even during Pleistocene glacial phases. Considering that lineage C showed a lack of geographic structure and that the highest genetic diversity of this lineage occurs along the eastern edge of its range, survival of this lineage in coastal or peri-coastal portion of the Padano–Venetian plain throughout the Pleistocene appears particularly plausible. A similar scenario was hypothesized previously on the basis of phylogeographic data for two other temperate amphibians, the Italian tree frog, *Hyla intermedia* [88], and the pool frog, *Pelophylax lessonae* [89]. Interestingly, this phylogeographic concordance, indicating long-term survival of essentially thermophilic taxa within the Padano–Venetian plain, suggests that a major glacial refugium for Mediterranean biodiversity just between the well-known Apennine and Balkan refugia could have passed mostly unseen for a long time. The post-glacial reflooding of the south-eastern portion of the Padano–Venetian plain likely erased most of the genetic imprints of such a refugial range in many species. Nevertheless, with an appropriate sampling scheme, such imprints could still be found in other species. This issue merits further research, even considering the growing interest in peri-coastal lowlands as glacial refugia and biodiversity hotspots in many regions worldwide (see [19], and literature therein).

### One species, three Pleistocene evolutionary histories III: Multiple refugia in peninsular Italy

In contrast to the northern lineages, lineage S showed a clear phylogeographic structure with five main subclades. Three of these subclades (SIII, SIV, and SV) occurred in the northern and central portions of the Calabrian peninsula, an area documented as a major hotspot of intraspecific biodiversity and as a site of multiple refugia for an increasing number of species [10], [11], [21], [43], [46], [88], [89], [106]–[108]. The other two subclades (SI and SII) occurred along the remainder of the Italian peninsula. The phylogeographic discontinuities between these latter subclades are located near the Volturno–Calore river drainage basin, another area of clustering of phylogeographic breaks and secondary contact zones for several species and intraspecific lineages (e.g. [46], [108], [109]). For both the Calabrian and the Volturno–Calore areas, glacio-eustatic sea-level oscillations throughout the Pleistocene and consequent insularization of southern Italy during multiple interglacial transgressions have been indicated as the most likely source of historical barriers to the dispersal of terrestrial fauna [10], [11], [21], [88], [106]. According to ecological and phylogenetic studies no evidence exists to support a sea crossing, even of modest distance, by this species or its close relatives. Thus, a scenario of palaeoinsularization as the source of the observed phylogeographic pattern appears plausible for *T. carnifex*.

The occurrence of five genetically divergent and geographically separated sub-clades within lineage S and the clustering of their estimated divergence times early in the Middle Pleistocene (Figure 2) suggest that the species survived most of the Pleistocene climatic oscillations within multiple separate refugia. This pattern is emerging in an increasing number of temperate species from the Italian peninsula [10], [11], [21], [43], [46], [88], [89], [106]–[108]. Nevertheless, contrary to most species studied to date, *T. carnifex* is currently absent from the south-central and southern portion of the Calabrian region, the hotspot of genetic diversity and the area richest in distinct refugia and divergent lineages in most of the studied species. Furthermore, contrary to findings from these previous studies, the ancient derivation of subclade SI (about 1 My; see Figure 2) suggests a long-term refugium in the northern portion of the peninsula rather than a post-glacial recolonization of this area from the south (see also [88]). Interestingly, recent updates of palaeoenvironmental data for the north-western peninsula (particularly the Arno river basin) indicate the presence of areas of prolonged ecological stability along the coastal plains [110], which could have acted as a glacial refugium for both plant and animal temperate species (see also [20], [88], [110]).

## Conclusions

The growing number of phylogeographic studies concerning the western Palaearctic region shows that the species' responses to Pleistocene climatic oscillations can be surprisingly diverse. The Italian crested newt, *Triturus carnifex*, exemplifies several of these species' responses. Multiple sources of evidence suggest that the three main lineages of this species have had substantially independent evolutionary histories in three distinct geographic districts throughout the Pleistocene, showing differential responses to Quaternary climate oscillations. Such responses encompass survival in a northern cryptic refugium (lineage N) and in peri-costal refugia (lineage C and sublineage SI), as well as in multiple refugia spanning most of the Italian peninsula (lineage S). These different regional responses also suggest diverse historical demographic trends for each lineage, which merit further investigation by using multi-locus data including information from variable regions of the nuclear genome. In addition, although different regional responses may be attributable primarily to the availability of suitable habitat through time associated with physiographic features and palaeogeographical histories, they may also be due to differences in lineage ecologies, which affect individualistic reactions to local factors [111], [112]. The development of lineage-specific ecological niche models will allow testing of whether distinct evolutionary lineages within this species have different niche associations and thus, unique responses to past and future climatic shifts [112].

The finding of three ancient and independent evolutionary units within the Italian crested newt also has important conservation implications. This species is currently listed as Least Concern on the International Union for Conservation of Nature (IUCN) Red list of Threatened Species ‘in view of its wide distribution, tolerance of a broad range of habitats, presumed large population’ [113]. However, our results do not fit this assessment and suggest that the three lineages should be considered distinct conservation units (ESUs; sensu [114]). Furthermore, lineage S appears fragmented into several population units of historical derivation that should be considered demographically independent (see [94]) for management purposes also (MUs; sensu [114]).

Finally, the importance of an appropriate sampling design in phylogeographic studies has been emphasized recently, and for temperate species of the western Palearctic special attention has been given to the southern portion of the species' ranges [7], [11], [15], [81]. The case of *T. carnifex* presented here reinforces this claim, and indicates substantial variation in the southern range of the species that would have passed unseen otherwise. This study also clearly shows that northern portions of peninsular species' ranges cannot be overlooked when developing sampling strategies, especially when our data are compared to those of previous assessments of genetic variation in this species.
